# Automatic Detection of the Orientation of Strain Gauges Bonded on Composite Materials with Polymer Matrix, in Order to Reduce the Measurement Errors

**DOI:** 10.3390/polym15040876

**Published:** 2023-02-10

**Authors:** Alexandru Serban, Paul Doru Barsanescu

**Affiliations:** Faculty of Mechanical Engineering, “Gheorghe Asachi” Technical University of Iasi, Blvd. D. Mangeron 43, 700050 Iasi, Romania

**Keywords:** CFRP composite, directional orientation, computational modeling, non-destructive testing, strain gauge, measurement error

## Abstract

Composite materials with a polymer matrix are used on a large scale to make light structures that involve high responsibility. The failure mechanisms of composite materials are very complex and for this reason, advanced techniques for damage detection and the assessment of structural integrity are required. The continuous structural health monitoring (SHM) uses nondestructive testing (NDT) techniques, sensors integrated into the structures, computers and dedicated software. This article presents a new automatic and precise method for detecting the orientation of strain gauges glued onto composite materials with a polymer matrix. The automatic identification of both the directions of the reinforcing fibers and that of the orientation of the strain gauge, respectively, allows for the calculation of the angle between these two directions. By knowing the difference between the nominal value of this angle and the value actually obtained after gluing the strain gauge, corrections obtained by calculation on the experimental values can be applied, using equations found in specialized literature. In this way, a drastic reduction of measurement errors introduced by the misalignment of strain gauges glued on composite materials can be achieved, resulting in a significant increase of measurement accuracy, which contributes to increasing the security of the monitored structures.

## 1. Introduction

Due to their special characteristics, long fiber-reinforced polymeric composite materials are widely used in different fields: aerospace, wind turbines, automotive, ship structures, civil construction, pressure pipes and vessels, biomedical, sport equipment, and so on. Military aircraft, such as the B2 bomber, have bodies made almost entirely of composite materials. Civil airplanes use composites particularly for fuselage and wings and these materials account for more than fifty percent of their weight. The composite industry appears to be ready for a rapid expansion in the future [1,2,3,4]. However, it is particularly difficult to detect early structural damage in composite materials. For this reason, careful monitoring of high-responsibility structures made of composite materials with polymer matrices is necessary in order to avoid catastrophic losses. Monitoring structures made of composite materials is a sure way to reduce costs and increase safety. Although visual monitoring is useful, such monitoring can only detect large surface damage, cannot be permanent and is subjective, imprecise and time-consuming.

The most used methods for structural health monitoring (SHM) are: strain gauges, optical fibers, digital image correlation (DIC), eddy current, acoustic emission, Lamb waves, modal analysis, piezoelectric, X-ray radiography and ultrasonic [5,6,7]. However, a continuous on-site monitoring of the structures can only be achieved using sensors, a computer and dedicated software.

In experimental mechanics, the strain gauge method is one of the most popular methods used to measure strain due to its versatility, low cost, simple procedures, etc. These sensors can be easily glued onto the surface of samples or structures or they can be embedded inside the structure of composites with polymer matrices. Strain gauges are useful for determining the elastic characteristics of materials (moduli of elasticity and Poisson ratios) or for determining residual stresses [8], etc. Additionally, strain gauges mounted on structures for the purpose of full-scale testing or monitoring can be used in order to determine the strain and stress in the area in which they are mounted, detecting and monitoring the presence of cracks [9] in order to determine the stress gradients in the vicinity of the stress concentrators, as indicators of material fatigue, etc. In recent years, a series of studies have been carried out on the monitoring of structures made of composite materials. Many of these have used the strain gauge method [10,11,12,13,14,15,16,17] or a combination of methods [18,19].

In general, composites reinforced with long fibers are orthotropic materials and only those reinforced in many directions are quasi-isotropic. Contrary to isotropic materials, the physical properties (Poisson’s ratio, modulus of elasticity, strength, etc.) of anisotropic and orthotropic materials are direction-dependent. Of course, both materials and finished products (components or structures) must be tested experimentally in the laboratory or in the field. In the case of semi-finished products (usually sheets or plates), the main directions of the material are marked on a label and pasted on. Different parts can be extracted from sheets or plates in order to make specimens or components. In the production process, larger portions from sheets or plates may remain. They can be used to make other components, but no longer have a label indicating the main directions. Unfortunately, in some composites, the main reinforcement directions cannot be easily observed with the naked eye.

The mechanical and elastic characteristics of fiber-reinforced polymer materials depend on the type of fibers, their percentage, their arrangement and their orientation. Extrapolating the experimental results obtained from carbon fiber reinforced polymer (CFRP) samples, the authors of the article [20] state that, for a plate with infinite width, “the strength decrease due to a 5° misorientation was approximately 20%.” For this reason, the precise measurement of fiber orientation plays an important role in quality assurance, both during the manufacture of composites and the realization of structures made of composite materials.

Several methods are used to determine the direction of the long reinforcing fibers of composite materials with polymer matrices, such as high-frequency eddy current imaging, X-ray (including computed tomography) and digital Image correlation (DIC) [21,22,23,24]. These methods use sophisticated and expensive equipment, whose use is restricted to laboratory environments. In addition, they have limited applicability; the eddy current method can only be used on conductive materials (including CFRP), DIC requires access to the composite material cross section, X-rays require access to both sides of the structure and present a safety hazard. In order to determine the orientation of fibers from composite materials, automatic detection methods are being used more frequently, because they ensure increased precision and decrease the amount of time required for operation. Many of these methods require sophisticated and expensive equipment, as well as signal processing techniques, statistical and/or artificial intelligence modeling, etc. [25,26,27,28]. An automatic method for the detection of fiber orientation on composite laminates using convolutional neural networks is presented in papers [29,30]. This method does not require sophisticated equipment, is cheap, quite fast and has high accuracy. In this article, references to the determination of fiber orientation will also be made. For this purpose, the method described in [29,30] will be used because both methods, the one used to determine the orientation of fibers and the one used to determine the orientation of strain gauges, respectively, use the same equipment and different, but compatible, software.

Due to the orthotropic behavior of the composite material, it is essential to know its main directions and to accurately align the strain gauges in relation to them, in order to correctly measure the strain [31,32]. The installation technology of strain gauges is well known [33,34]. This involves, inter alia, applying the markings on the tested component for visual alignment with marks existing on the strain gauges support. Thereby, the positioning of strain gauges is inevitably done “by eye measurement” and the accuracy depends on the skill of the person who installs the transducer. The most used strain gauges, both on metals and on composites, have grid lengths of 6 mm and 10 mm [32].

A rotation of the strain gauge relative to the direction on which the measurements are wanted introduces misalignment errors. For the above transducers, these errors are up to ±5°, for the experienced operator working in ideal conditions [31,32,33,34,35,36]. The magnitude of the error caused by misalignment depends upon the following factors: the ratio of the maximum and minimum algebraic strains σ_p_/σ_q_, the angle between the maximum principal strain axis and the intended axis of strain measurement and the angular positioning error between the glued transducer axis and the strain axis that is intended to be measured [37,38,39]. Mathematical corrections for the misalignment of the strain gauges can be made using well known equations of stress at a point for isotropic materials. Thus, a misalignment of 5° for a strain gauge glued on isotropic metallic materials generally introduces fairly small errors, until 1.5% [31,35,36]. However, for a misalignment of 5° of a T rosette glued on a pressurized cylinder with an axial compressive load, a −0.68% error in σ_p_ and a 6.75% error in σ_q_ is reported [33]. According to [40], the change of sensitivity due to misalignment is 0.88%/με/mm for strain gauges glued on a stainless steel cantilever subjected to bending.

It should be noted that the same misalignment error produces a much larger error in strain measurement on the orthotropic materials than on the isotropic ones. For uniaxial state of stress (with ε_x_ = 0.5%) and 5° misalignment of the strain gauge glued on different orthotropic laminates, the following relative measurement errors are reported: 20% for UD CFRP [0°]_4sym_ and 6% for UD GFRP [0°]_4sym_, compared to 1% for quasi-isotropic laminate [−45°, 0°, +45°, 90°]_sym_ [32,41]. In some cases, the misalignment of strain gauges may introduce errors exceedingly, even 50% in strain measurement [37]. Moreover, for measurements conducted in the field or on large structures, it is more difficult to assure a misalignment error of only 5° and, as a consequence, the measurement errors will be even bigger. Of course, such measurement errors cannot be accepted. The only solution to increase the accuracy is to correct, by calculus, the measurement results [42,43,44]. To this end, “considerable care must be given to both identification of the principal material directions and to strain gage alignment” [37].

By accurately knowing both the main directions of the composite material and the direction of the strain gauge filaments glued on it, orientation errors can be substantially reduced using corrections by calculation. Thus, the precise determination of these two directions and the angle between them is of major importance in the case of precise measurements with strain gauges glued on composite structures. Strain gauge orientation can be visually determined. With the naked eye, the method is imprecise, subjective and time-consuming. Assisted by an optical microscope, the method can give much more precise results, but it is also time-consuming. It may be assumed that it is quite difficult to use an ordinary microscope on large structures. Both the orientations of the reinforcing fibers and of the SG, respectively, can be determined by the X-ray method. This method uses complex equipment, which requires access to both sides of the structure and could be dangerous for people’s health.

In the paper [29], it is shown that the fiber orientation of composites can be automatically detected with good accuracy based on classical image processing and computational geometry. Another method can do the same using convolutional neural networks [30]. It turns out that these methods give good results, even for cases where the orientation of the composite’s fibers can be observed with difficulty with the naked eye.

Starting from the bibliography above, this article presents a new method for the automatic detection of the orientation of strain gauges glued on composite materials. After the automatic detection of direction of the reinforcing fibers and the orientation of the strain gauge glued on composites, the angle between the two directions can be determined and corrections can be made by calculation, which leads to the reduction of measurement errors due to the misalignment of strain gages. The proposed method has high accuracy. The visual detection of the misalignment angle of a strain gauge is imprecise and time-consuming.

## 2. Method Description

The method proposed for the automatic detection of the angle between the main direction of reinforcement of a composite material and the direction of strain gauge (SG) filaments combines the use of neural networks with classical image processing techniques and computational geometry.

The main stages that form the basis of the proposed method are listed below and will later be described in detail:

1. Detection of SG positioning within the acquired image, in the form of a binary mask representing the connected domain associated with the transducer;
2. Detection of the orientation of the SG filaments (θ_SG_) with the help of the existing triangular markings on the transducer support, which indicates the parallel and respectively perpendicular directions in relation to the transducer filaments;
3. Detection of the orientation of the reinforcement fibers of the analyzed composite material (θ_fibres_);
4. Calculation of the angular positioning error β as the deviation between θ_SG_ (determined in step 2.) and θ_fibres_ (determined in step 3).

### 2.1. Automatic Detection of SG Positioning

Following the implementation of this procedure, it is desired to obtain the exact positioning of the transducer within the analyzed image, in the form of a binary mask. The formulation of this problem is a classic one in the field of image processing and there are a multitude of methods that can be used to solve it. For example, classical image processing based on intensity, gradients and color histograms can be applied to extract the region of interest. The main advantage of such an approach would be the simplicity of implementation, while disadvantages include accuracy and stability that may degrade if the transducers and the composite material have similar chromatic properties. For the same purpose, complex neural methods can be used for semantic segmentation, such as CMX and DeepLabV3+ [45,46], based on a convolutional architecture. The advantages of such neural models are represented by the precision and stability of the results, and among the disadvantages, we can mention the complexity of the implementation in practice; generating the necessary data sets involves a consistent effort, the neural architecture is complex, etc.

Given the above observations, the following objectives were considered in the development of the automatic SG positioning detection method:

- *Simplicity of implementation*—the method must be easy in order to be implemented in practice;
- *Accuracy and stability*—the results obtained with the developed method should be optimal, even when the transducer–composite chromatic configuration is difficult;
- *Speed*—the detection time of the region occupied by the SG must be as short as possible;
- *Reduced complexity*.

As a result of the objectives presented above, the decision was made to implement a method based on a very simple neural model, capable of determining whether a small-sized region (2r+1)×(2r+1) is associated with SG or with the composite material. Typical sizes for classified regions are 5 × 5 or 7×7, corresponding to values of 2 and 3 for the radius r, respectively. Moreover, the details related to the architecture of the proposed neural model, the generation of the datasets used to train and evaluate the model, as well as the performances obtained when classifying small regions using the neural model will be presented. Subsequently, the automatic method of extracting the SG positioning from the acquired image, using the neural classifier, will be described in detail.

The architecture of the neural model is as follows:

- The input layer is represented by a vector containing the flattened region of small dimensions (2r + 1) × (2r + 1) to be classified as belonging to SG or to composite material. For an RGB image and a typical value for r = 3, this results in an input vector size of 147. The input vector is also normalized by dividing with 255;
- Two fully connected layers were used as hidden layers with ReLU (*rectified linear unit*) activation, Equation (1)
  (1) ReLU(x)=max(0,x)
  having 256 neurons;
- The output layer consists of a single neuron with sigmoid activation that provides the probability of the analyzed small region belonging to the transducer.

The *optimized function* is *binary cross-entropy,* which has the following form:

(2) $$H=-\frac{1}{N}\sum_{i=1}^{N}\left[y_{i}\log\;{\hat{y}}_{i}+(1-y_{i})\log(1-\;{\hat{y}}_{i})\right]$$

where N is the number of observations in the minibatch, ${\hat{y}}_{i}$ is the estimated probability that the region associated with the ith observation in the minibatch belongs to the transducer, and $y_{i}$ represents the correct value for the ith observation.

Minimization of the cost function is done using *minibatch stochastic gradient descent* (mSGD) with momentum (Equations (3) and (4))

(3) $$V_{t+1}={\mu V}_{t}-\alpha \nabla_{\theta }L\left(x,\theta \right)$$

(4) $$W_{t\;+\;1}\;=\;W_{t}\;+\;V_{t\;+\;1}$$

where μ is the momentum, α is the learning rate, L(x, θ) is the optimized function and W represents the parameters learned by the model.

The proposed neural classifier was implemented in *Tensorflow* GPU. The computing station used for training and inference has the following relevant specifications: i7 6700HQ CPU, 16 Gb RAM, nVIDIA 950M GTX GPU.

**Figure 1:**
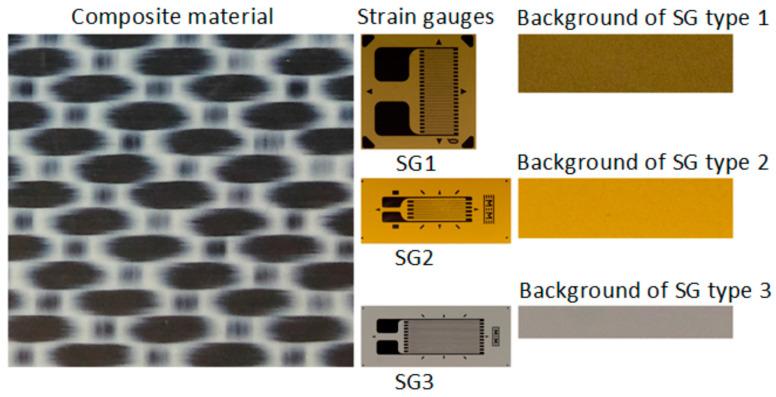
Sample of *initial images*.

The dataset generation procedure is very simple and consists of generating the *input images* for the neural model, starting from *initial images* with the composite material and, respectively, with background regions of the analyzed transducers. Some examples of initial images are shown in Figure 1, where the images corresponding to the transducers are shown only to put into context the *initial images* corresponding to the SG backgrounds. Full transducer images are not used to generate datasets.

The reason initial images containing only the background of the transducers are used is related to the precaution of not generating confusion when training the neural model.

Since the colors of the markings on the transducers can resemble the colors of some areas on many composite materials, and the regions selected as *input images* are very small in size, there is a risk that randomly generating the input images from the *initial images* will result in input images that are chromatically similar to the composite material and the transducers, which would be inconsistent for training the neural classifier.

Three disjoint data sets were used to train and evaluate the model: the *training* set, the *validation* set, and the *test* set. The model is trained using the training dataset and then evaluated using the validation dataset. If necessary, model parameters are hyper-tuned, then retrained and re-evaluated using the training and validation datasets, respectively. This process is iterated until the desired performance for the validation set is achieved. Because the validation set is used several times in the iterative process described above, and in order to avoid reporting generalization performances influenced by hyperparameter tuning, a third dataset will be used, namely, the test dataset.

The first step in generating datasets for training, validation and testing is to randomly split the initial acquired images as follows: 80% for training, 10% for validation and 10% for testing. In order to generate a single input image, for any of the three data sets, an initial image corresponding to that data set is randomly selected, and from this initial image, a region of the image dimensions (2r+1)×(2r+1) is used, which, after flattening, will represent the input vector for the neural model. If the size of the original image is w × h, then (w−2r)×(h−2r) distinct input images can be generated from this image. Since the typical values of *r* (e.g., 2 or 3) are much smaller than those of w and h (which are in the hundreds or even thousands), it thus results that many distinct input images can be generated from a single *initial image*—of the order of tens of thousands or even hundreds of thousands. This remark is intended to emphasize the fact that, following the generation procedure, robust data sets with many observations are very easily obtained, starting from a small number of initial images.

Unlike the *initial images* corresponding to the SG background, which are uniform, the *initial images* corresponding to the composite material have repetitive motifs, and in order to obtain a neural classifier robust to variations in the orientation of the composite material fibers (repetitive motifs) it is necessary to include these variations in the datasets. This is easily done by adding an intermediate step after selecting the *initial image*, which consists of rotating it by an angle whose value is randomly generated. Note that this intermediate step is only used for the *initial images* associated with the composite material.

A final comment would be related to the random selection of the *initial image* used to generate the *input image*. The selection is made according to a discrete distribution where the selection probabilities are proportional to the number of distinct *input images* that can be generated from the *initial images*. The training of the proposed neural model is done in minibatches of size N = 256, for 50,000 iterations, with a learning rate α = 0.05, the duration being about 7 min. The obtained classification accuracy on the test data set is 99.9891% and this performance is not surprising because the chromatic difference between the composite material and the SG background is quite large and, consequently, the classification problem is relatively simple.

**Figure 2:**
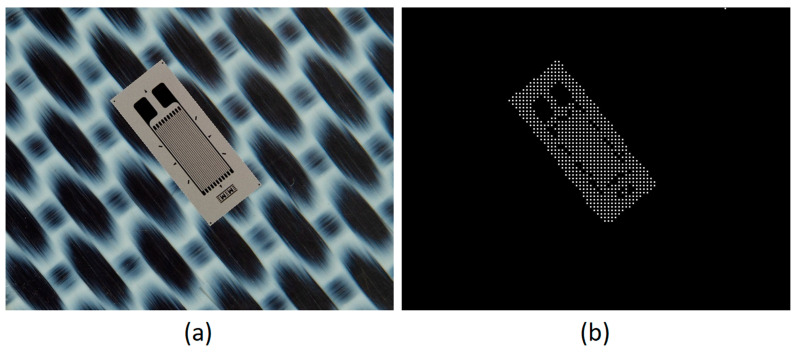
Acquired image (**a**) and output (**b**) from step 1 processing.

**Figure 3:**
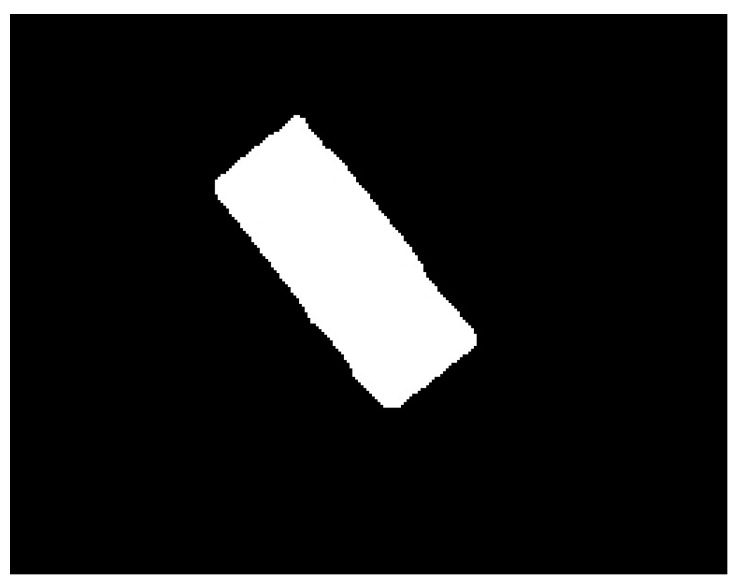
Result obtained after the processing in step 2.

The neural model presented above is used as the main element in the automatic SG positioning detection procedure. The main steps of this procedure are described and illustrated as follows:

1. Equidistant positions are generated both horizontally and vertically for the acquired image containing the composite material with the attached transducer. The distance between these positions is controlled by the parameter *s*, which has typical values such as 5, 10, 20. For each position, a region of dimensions (2r + 1) × (2r + 1) is selected, which is subsequently classified as belonging to the composite material or to the transducer, using the neural model described in this section. Depending on the classification result, that position is associated with either the composite material or the SG. The purpose of this step is to quickly find an approximate positioning of the transducer within the image. If every possible position within the image were evaluated (situation equivalent to setting the parameter *s* to the value 1), the SG mask would be obtained directly from this step. However, using this strategy, the computational effort would be huge, since many classifications with the neural model are required. If the value of the parameter s is greater than 1, then the number of classifications becomes s^2^ times smaller. Figure 2 shows the acquired image and the result obtained after this phase;
2. Interconnect the positions classified as belonging to SG in step 1 by marking the rectangular region associated with a position. For a position classified as SG, the rectangular region bounded by the eight adjacent positions is colored only if all adjacent positions are classified as belonging to SG. Following this procedure, a simply connected domain is expected to result, representing a raw mask for the transducer. If several connected domains are obtained, as a result of possible classification errors with the neural model, only the one with the largest area is kept. Additionally, for the selected domain, any gaps, possibly generated by the markings on the transducer, are filled. Figure 3 shows the result obtained after this phase;
3. For the image that represents the raw mask, the following processes are successively applied:
  (a) *Erosion*—a rectangular filter of size (2s + 1) × (2s + 1) is used in order to obtain a shrunk version of the SG mask;
  (b) *Dilation*—a rectangular filter of size (4s + 1) × (4s + 1) is used in order to obtain an enlarged version of the mask;
  (c) XOR—is performed between the eroded image obtained in step 3.a and the dilated image obtained in step 3.b.

**Figure 4:**
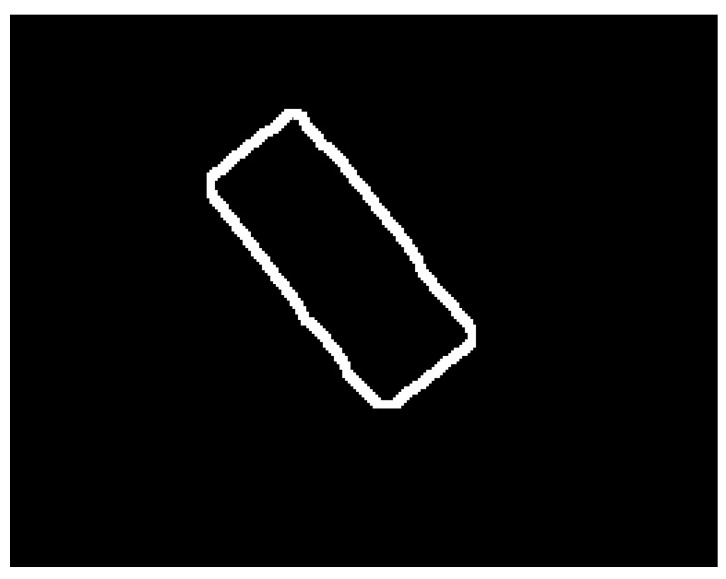
Result obtained after processing from step 3.

**Figure 5:**
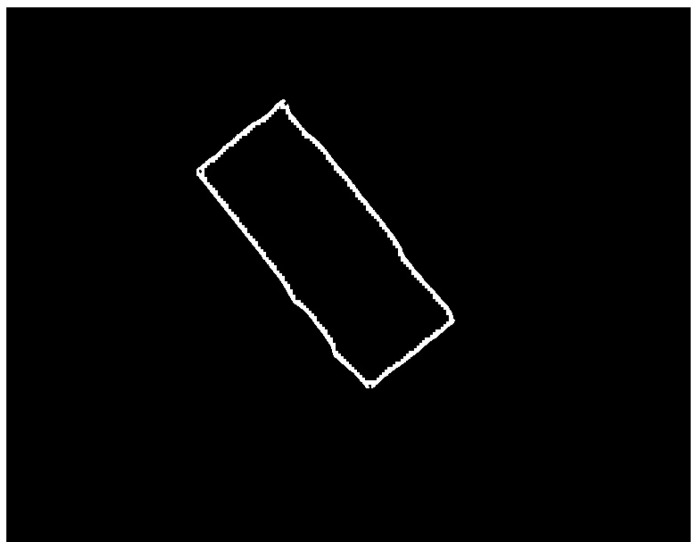
Result obtained after processing from step 4.

The purpose of the processing in this step is to obtain a coarse band within which the chromatic border of the transducer is found. Figure 4 shows the result obtained after this phase;

4. For all positions contained in the coarse band obtained in step 3, the neural model is used to classify them as belonging to the composite material or to the transducer. Following this processing, it is expected that the coarse band obtained in step 3 will erode so that the outer contour of the new (fine) band accurately follows the chromatic contour of the transducer. Figure 5 shows the result obtained after this phase;
5. Finally, the following post-processing is applied to the image obtained in step 4:
  (a) Filling the domain bounded by the fine band, thus obtaining the SG mask;
  (b) Extracting the outline of the SG mask as a polygon.

**Figure 6:**
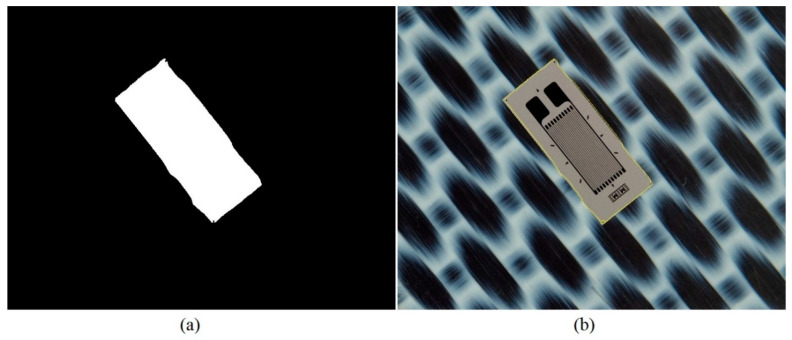
Final results obtained from post-processing in step 5: (**a**) SG mask and (**b**) SG contour (yellow) superimposed on the acquired image.

The final results of the procedure are presented in Figure 6.

As can be seen from the results obtained with the automatic SG positioning detection procedure, the transducer is identified with very high accuracy. The transducer model used to illustrate the positioning detection procedure was chosen so that it has chromatic characteristics close to those of the composite material in order to stress the proposed method as much as possible. Additionally, the execution time of the procedure is very small, about 0.2 s (on the computing station specified in this section), due to the optimizations, according to which, the number of predictions/queries using the neural classifier is drastically reduced. The automatic SG positioning detection procedure is a demonstration where classical image processing techniques harmoniously blend with neural models in the field of artificial intelligence.

### 2.2. Automatic Detection of SG Filament Orientation

The orientation of the SG filaments is determined with the help of the four triangular markings, respectively being in the direction parallel to the filaments, and the other two being respectively in the direction perpendicular to the filaments. The main steps underlying the determination of filament orientation are listed below. Subsequently, they will be described in detail and illustrated with images and graphs that represent the intermediate results obtained with the proposed method:

1. In order to determine an approximate orientation of the SG, the minimum area enclosing rectangle (MAER) is calculated. It contains the mask resulting from the SG position detection step. The slope of the long side of the MAER (whose angular orientation with respect to the horizontal axis is denoted by θ_MAER_) is used as the orientation characteristic;
2. Only that part of the image contained by the minimum area rectangle detected in step 1 is extracted and rotated around the center by the value of θ_MAER_. The result of this operation is that the image processed in the following steps contains only the SG at an approximately horizontal orientation;
3. A binary image is obtained in which the triangular markings, 45° markings, SG filaments etc. are represented;
4. Objects are extracted from the binary image and their outline is determined. Additionally, in this phase, various dimensional filters can be applied in order to eliminate those objects about which can be said with certainty that they are not triangular markings;
5. Triangular markings are determined. Having an approximately horizontal orientation of the SG, the relative positions of these markings can be easily identified: *north*, *south*, *east* or *west*;
6. The angular orientation θ_HORIZ_ of the SG filaments is determined as the orientation of the straight line that passes through the centers of gravity of the triangular markings corresponding to the east and west relative positions;
7. Accurately calculate the initial orientation θ_SG_ of the SG filaments as the sum of θ_MAER_ and θ_HORIZ_.

As it can be seen from the description of the SG filament orientation determination procedure, this is a deterministic one, not using neural models like those applied in the automatic fiber orientation detection method [30]. There are two reasons for choosing this deterministic approach:

1. The chromatic characteristics of the region representing the SG are favorable in the sense that they allow for the markers present in this region to be easily detected. In other words, the binary image resulting from image processing using classical techniques is clean, stable and accurately reproduces the markers for SG orientation;
2. The availability of triangular orientation markings and determination of SG orientation based on them is indisputable, being similar to the procedure applied in practice for gluing SG. Another option would be to determine the orientation based on the filaments resulting from image processing, but this approach was more laborious, more prone to errors and required an image acquired at a very high resolution, given that the width of these filaments is very small.

Next, the steps of the proposed method will be detailed, and a stability analysis will be made where appropriate.

**Figure 7:**
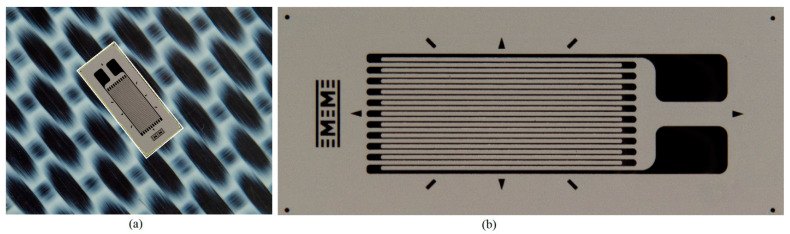
Image extraction of SG and orientation in approximately horizontal position: (**a**) phase 1 and (**b**) phase 2.

In step 1, the same method as described in Section 2.2.2 from [29], is used to calculate the MAER, except that the procedure is applied to the contour of the SG mask resulting from the automatic detection of SG positioning (step 5b). In step 2, the region in the acquired image corresponding to the MAER is extracted and rotated by a value equal to the angle between the large side of the MAER and the horizontal axis, θ_MAER_, in the anti-trigonometric sense, resulting in an approximately horizontal orientation of the SG. The application of these two stages is illustrated in Figure 7 for the type 3 transducer.

In order to obtain the binary image, several image-processing procedures are applied in step 3:

- *Luminance* calculation using the same method as described in Section 2.2.1 from [29];
- As optional processing, a mean filter (as described in Section 2.2.1 from [29]) or a Gaussian filter can be applied;
- Transformation of *luminance* into binary image based on pixel intensity, using the Otsu method [47,48];
- *Complementing* the binary image obtained in the previous step.

**Figure 8:**
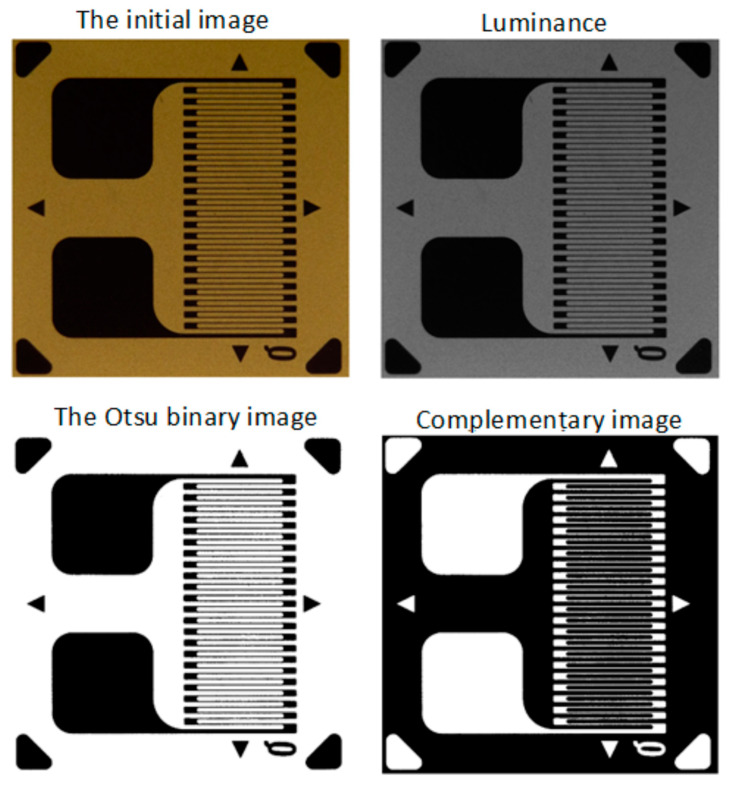
Obtaining the binary image for the type 1 transducer (step 3).

**Figure 9:**
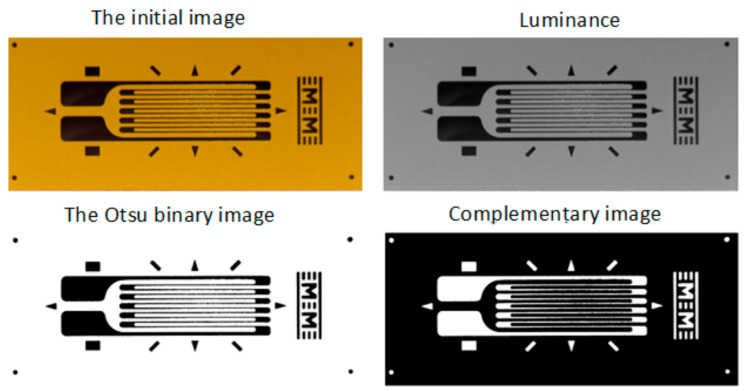
Obtaining the binary image for the type 2 transducer (step 3).

**Figure 10:**
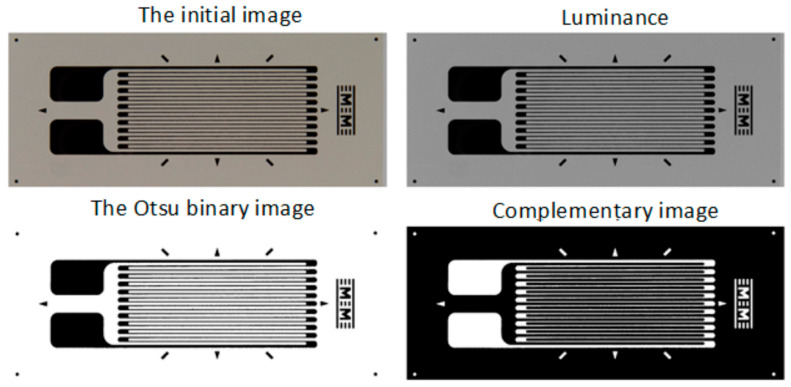
Obtaining the binary image for the type 3 transducer (step 3).

The successive processing of step 3 is illustrated in Figure 8, Figure 9 and Figure 10 for the analyzed transducers. It is easy to see from the figures that the resulting binary images have a very good quality, accurately representing the markings present on the transducers.

**Figure 11:**
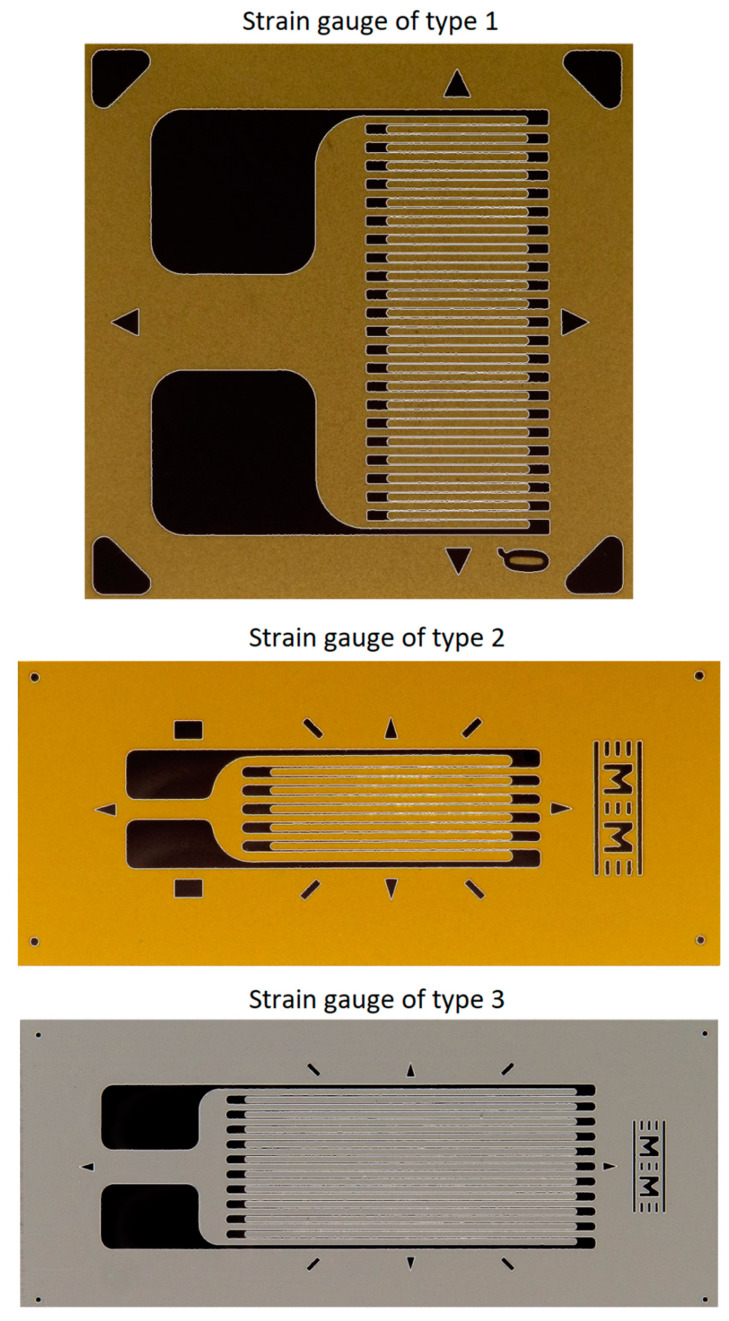
Extracting objects from the binary image and overlaying contours over the original image (step 4).

The extraction of objects from the binary image in step 4 is done using the same filling/flood-color procedure described in Section 2.2.2 from [29]. This phase is a buffer between image processing and geometric processing, because it takes an image as input data and provides as output data some sets of points in Euclidean space representing the objects and their boundaries. Dimensional filters can also be applied in this phase in order to eliminate those objects that are not of interest. Some examples of filters would be: those based on the *bounding box* dimensions of the objects, those based on the areas of the objects, etc. In Figure 11, the borders of the detected objects for the three types of analyzed transducers are illustrated in white. As expected, given the good quality of the obtained binary images, the borders of the detected objects faithfully follow the contours of the markings on the transducers.

In step 5, those objects that have a triangular shape are selected. The selection is made through a procedure that involves the analysis of the objects’ borders detected in phase 4. This procedure is described and illustrated as follows and involves:

- Calculation of the center of gravity of the boundary of the object, which is done with relations (11a,b) and (12) from Section 2.2.2 of [29].
- Developing the *distance profile,* which consists of calculating the distance from the center of gravity to each point on the object’s border, maintaining the order of the points;
- *Circular displacement of the distance profile* so that the first position corresponds to the minimum distance in the profile;
- *Iterative smoothing of the distance profile* in order to remove noise caused by imperfect detection and discrete space of point positions. For this, a filter window of size r is considered, and the distance value from a certain position i in the distance profile is replaced by the average of the distance values from position i − r to position i + r. This smoothing process is executed in an iterative manner by a number of times equal to *niter*;
- Detecting the minimum and maximum positions of the shifted and smoothed distance profile;
- An object is validated as triangular if the following conditions are met:
  o The *center of gravity* is inside the object’s border;
  o The *shifted and smoothed distance profile* has exactly three *minimum positions* (which would correspond to the midpoints of the sides of the triangle) and has exactly three *maximum positions* (which would correspond to the vertices of the triangle);
  o All distances corresponding to *minimum positions* are less than a value l_min_ and all distances corresponding to *maximum positions* are greater than a value l_max_.

**Figure 12:**
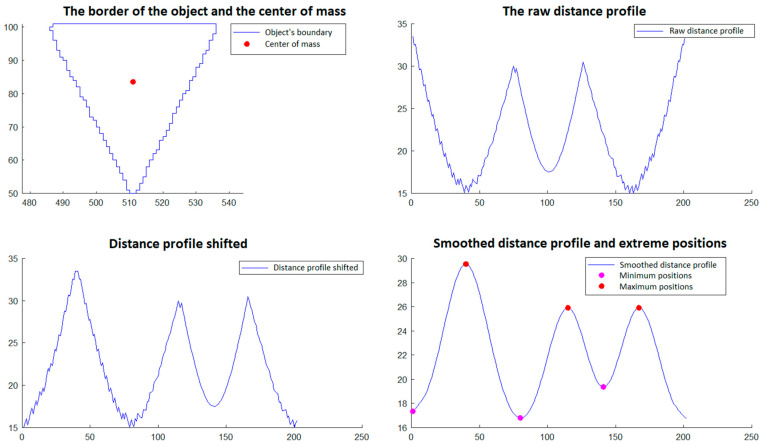
Triangular object selection procedure (step 5)—South triangular object.

**Figure 13:**
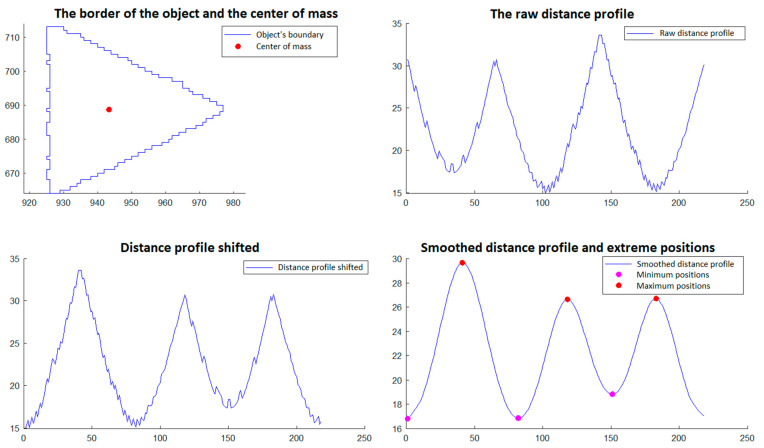
Triangular object selection procedure (step 5)—East triangular object.

**Figure 14:**
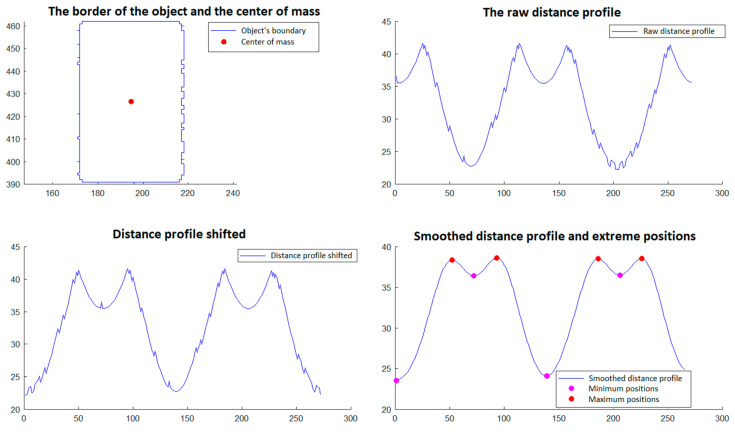
Triangular object selection procedure (step 5): non-triangular object.

**Figure 15:**
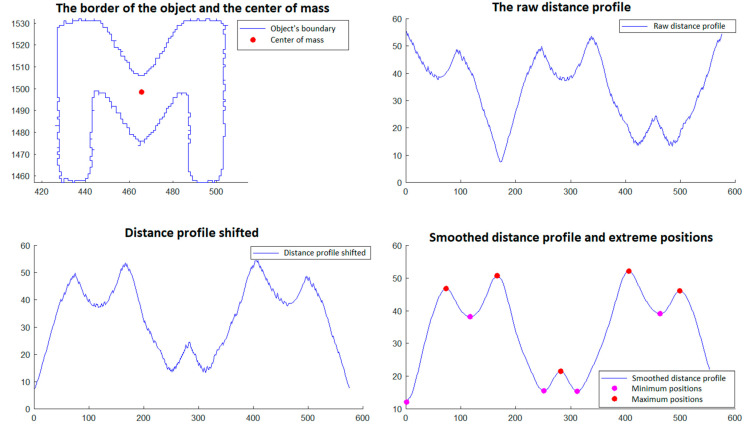
Triangular object selection procedure (step 5): non-triangular object.

The procedure described for selecting triangular objects is illustrated in Figure 12 and Figure 13 for two triangular objects and in Figure 14 and Figure 15 for two non-triangular objects. It is expected that following this procedure, four objects corresponding to the triangular markings will be selected for SG positioning. Since, in step 5, the transducer is in an approximately horizontal position, it is very easy to associate the triangular markings with the relative positions within the SG (*north*, *south*, *east* and *west*). For the *north* relative position the triangular mark that has the largest ordinate of the center of gravity is associated; for the *south* relative position, the triangular mark that has the smallest ordinate is associated; for the relative *east* position the triangular mark that has the largest abscissa of the center of gravity is associated, and for the relative *west* position, the triangular mark that has the smallest abscissa is associated.

In step 6, the angular orientation θ_HORIZ_ of the SG filaments is determined as the orientation of the line that passes through the centers of gravity of the triangular markings corresponding to the *east* and *west* relative positions. The value of θ_HORIZ_ is expected to be relatively close to 0°, since the SG has an approximately horizontal orientation after step 2 of processing. Additionally, the orientation of θ_VERT_ perpendicular to the SG filaments is determined as the orientation of the line passing through the centers of gravity of the triangular markings corresponding to the relative *south* and *north* positions. The purpose of determining θ_VERT_ is to validate/verify the consistency of the detection, the expectation being that the two lines on the basis of which the horizontal and vertical orientations are calculated are perpendicular. Below, we present the statistics obtained when validating the perpendicularity using one copy of each of the three types of transducers analyzed:

- Type 1 transducer: min = 90.1315°, max = 90.1724°, mean = 90.1541°, standard deviation = 0.0087°, obtained using 40 acquired images;
- Type 2 transducer: min = 89.9228°, max = 90.0009°, mean = 89.9779°, standard deviation = 0.0175°, obtained using 29 acquired images;
- Type 3 transducer: min = 89.9071°, max = 89.9381°, mean = 89.9252°, standard deviation = 0.0054°, obtained using 50 acquired images.

From the statistics presented above, it can be seen that there is a deviation of the order of 0.1° from the ideal value of 90°. However, the variations obtained for different images of the same transducer are approximately half, of the order of 0.05°. The deviations are extremely small and do not justify the effort to investigate their source in depth. The most possible causes for the appearance of deviations could be: the physical printing of the triangular markings on the analyzed transducers or the slight deformation of the image on the acquisition phase. A final aspect regarding step 6 would be the one related to using the centers of gravity of the triangular markings in order to determine the two directions. The reason the points of the triangles are not used rather than the centers of gravity is related to stability. Although the obtained binary image has a very good quality, there is still a risk of small disturbances appearing in the area of the vertices of the triangles that represent the markings. It is quite possible that these small perturbations do not influence the results of the algorithm. However, it is expected that the stability of the centers of gravity will be higher, because in their calculation, all the points on the border are used, reducing the influence of local disturbances.

**Figure 16:**
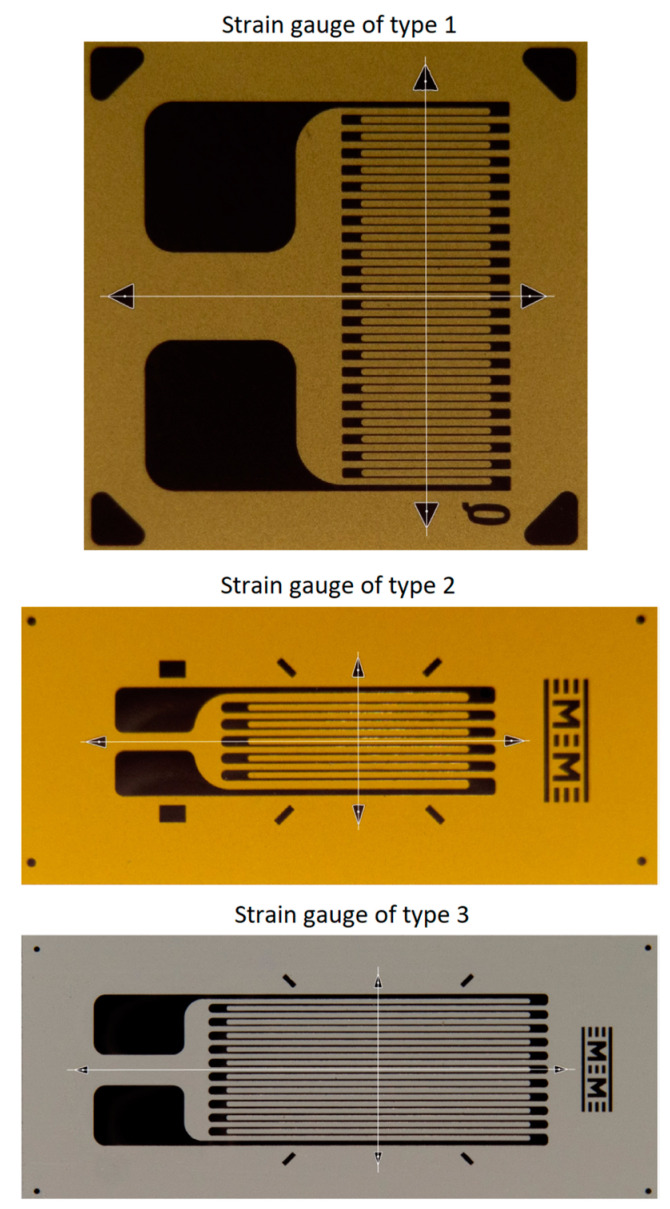
The directions parallel and perpendicular to the SG filaments, automatically detected.

Finally, in step 7, the precise orientation θ_SG_ of the SG filaments, for the initial unrotated image, is trivially calculated as the sum of θ_MAER_ and θ_HORIZ_. Figure 16 shows the results of detecting the direction of the SG filaments, as well as the direction perpendicular to them, for the three types of transducers that were analyzed using the algorithm presented in this section.

The parameters of the proposed method for the automatic detection of the direction of the SG filaments, in the case of the three types of transducers analyzed, are as follows:

- Type 1 transducer: distance profile filtering window of size r = 10, iterative filtering *niter* = 2 times, validation of minimum positions using l_min_ = 20, validation of maximum positions using l_max_ = 25;
- Type 2 transducer: distance profile filtering window of size r = 10, iterative filtering *niter* = 2 times, validation of minimum positions using l_min_ = 25, validation of maximum positions using l_max_ = 20;
- Type 3 transducer: distance profile filtering window of size r = 3, iterative filtering *niter* = 2 times, validation of minimum positions using l_min_ = 20, validation of maximum positions using l_max_ = 15.

### 2.3. Automatic Detection of Fiber Orientation of Composite Material

The detection of the θ_fibres_ orientation of the composite material’s fibers upon which the SG is mounted can be done using the method described in [29] (method 1) or the neural model described in [30] (method 2). The only precaution to be taken is to select one or more rectangular regions from the acquired image that contain only the composite material, without portions of the SG. This is easy to do since the SG positioning automatic detection procedure (section *Automatic detection of SG positioning*) results in a mask for the image region occupied by the transducer. Thus, the selection of rectangular regions, which are subsequently sent to be processed using method 1 or method 2 for the fiber orientation detection, is done taking into account that these regions do not overlap at all with the corresponding SG region.

Finally, the angular positioning error β of the SG is calculated as the difference between the orientation θ_SG_ of the SG filaments (determined in section *Automatic detection of SG filament orientation*) and the nominal orientation of the SG relative to the orientation θ_fibres_ of the composite material’s fibers (determined using either method 1, or method 2 for fiber orientation detection).

## 3. Conclusions

In the case of orthotropic materials (like most composite materials are) the angular orientation errors of the SG can be much higher than in the case of isotropic materials and cannot be neglected. Knowing precisely both the main direction of the material and the direction of the SG filaments, the angle between these directions can be determined; the orientation errors can be substantially reduced following the corrections by calculation known from the literature. Thus, the precise determination of these angles is of major importance in the case of precise measurements with strain gauges glued onto structures made of composite materials. Unfortunately, in the case of structures made of composite materials, there are sometimes major difficulties in establishing the reinforcement directions with the naked eye. The errors introduced when determining the angle between main reinforcement direction and direction of SG filaments can compromise the accuracy of the corrections by calculating the misalignment errors, which affects the output signal of strain gauges. In order to improve the accuracy of the measurements, an automatic method for detecting the direction of the strain gauges glued onto composites was proposed in this article.

The method presented for automatic detection of strain gauges (SG) orientation glued onto composite structures is a complex one compared to the two proposed methods for fiber orientation detection [29,30]. This is not surprising because the problem involves several steps: SG position identification, SG orientation detection, and fiber orientation detection. However, these phases are independent, which allows for the modularization of the overall solution. The best example of this is the use of method 1 or method 2 (proposed in [29] and [30], respectively) within the fiber orientation detection module. For these reasons, the complexity of the proposed solution for the automatic detection of SG orientation errors will not be considered as a weak point, but rather as a necessary fact in the context of the problem to be solved. The strengths and weaknesses of the automatic method of detecting the orientation of SG bonded to a composite material are presented below.

*Strengths*:

1. The computing station required for the practical implementation of the proposed solution does not involve special performance specifications.
2. The execution time of the procedure is reduced, being less than one second for the computing station on which the numerical experiments were run.
3. The proposed solution is modular and involves the following main steps: identification of the position of the SG glued on the composite material, detection of the orientation of the SG and detection of the orientation of the reinforcing fibers of the composite. This allows for any improvements made to one of the modules to be very easily integrated into the overall solution, without affecting the other modules.
4. The method is stable and accurate, as demonstrated by the analyses done throughout this paper.
5. Neural models are used in the SG position identification and fiber orientation detection steps, thus eliminating the tedious parameter calibration process present in classical image processing approaches. Additionally, neural models have simple architectures and are easy to reproduce in practice.
6. Although a classical deterministic procedure is used in the SG orientation detection step, it is controlled by few parameters and their calibration is very easy.
7. Neural models can be easily scaled if accuracy improvement is required, both horizontally (by increasing the number of neurons and convolutional filters) and vertically (by adding new neural layers).
8. Although complex neural architectures specialized for semantic segmentation, such as CMX and DeepLabV3+ [45,46], could be used for the SG position identification step, the problem was reformulated so that a very simple neural model could be used without performance degradation. This was motivated, in particular, by the need to easily generate datasets. Otherwise, the method would have become impractical.

**Figure 17:**
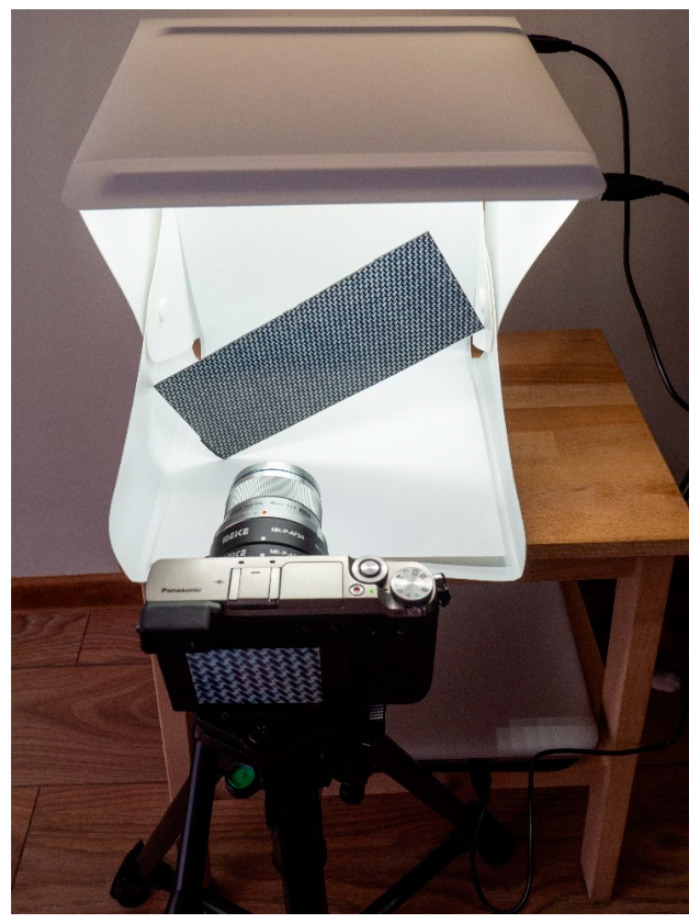
The image acquisition environment used in the automatic detection of SG orientation errors (photographing the composite board).

*Weaknesses*:

1. The hardware support required for image acquisition must be professional due to the small size of the strain gauges. For example, the filament lengths for type 1, 2 and 3 transducers used in the experiments in this article are 3.1 mm, 5.2 mm and 6.7 mm, respectively. In order to capture the necessary details, especially in the SG orientation detection step, a very good resolution and a good quality image is required, without reflections or other artifacts that could destabilize the procedure. Figure 17 shows the environment in which the images used in the automatic detection of SG orientation errors were acquired. Since the experiments undertaken throughout this paper are relatively few, issuing recommendations/specifications for a possible image acquisition system is difficult. This aspect is very important, and the analyses presented for the proposed method are, at this moment, insufficient to allow the project to advance from the research phase to the finished product phase applicable in practice, at least in terms of hardware support for image acquisition.
2. Neural models must go through a training stage. However, the dataset generation procedure is clear and easily reproducible for both the SG positioning identification step and the fiber orientation detection step (if method 2 presented in [30] is used). Furthermore, training of fully connected and/or convolutional networks is done quite quickly, within minutes, depending on available hardware support (GPU or CPU).
3. The method can only be used for strain gauges glued on the surface of the structures and it cannot be used for strain gauges embedded in the composite structures with polymer matrices.

## Data Availability

Data will be made available on request.
